# Cardiac CapZ Regulation During Acute Exercise in Female Mice

**DOI:** 10.1096/fj.202502431R

**Published:** 2025-08-20

**Authors:** Logan K. Townsend, David Wright, W. Glen Pyle

**Affiliations:** ^1^ Department of Human Health and Nutritional Sciences University of Guelph Guelph Ontario Canada; ^2^ Women's Health Research Institute Team at BC Women's Hospital + Health Centre Vancouver British Columbia Canada; ^3^ IMPART Team Canada Investigator Network Saint John New Brunswick Canada

**Keywords:** actin capping protein, acute exercise, heart, myofibrils, Z‐discs

## Abstract

Exercise requires a rapid cardiac response to maintain cardiovascular function. CapZ is a critical stress‐response protein in cardiac myocytes. While its role in the pathological stress response has been explored, its part in the physiological response to exercise is unknown. This study examined CapZ regulation during exercise to determine its importance in the cardiac response. Female wildtype or cardiac CapZ‐deficient transgenic mice (“CapZ mice”) were subjected to exhaustive swimming or running protocols and submaximal running. Time to exhaustion was a measurement of exercise capacity. Following submaximal exercise, cardiac myofilaments were isolated and probed for CapZ, its regulatory proteins, and myofilament proteins. Myofilament function was assessed using an actomyosin MgATPase assay, and protein phosphorylation was quantified with ProQ Diamond staining. Total myofilament CapZ was unaffected by exercise, but increased CapZIP and decreased phosphorylated CapZIP indicated weakened CapZ‐actin interaction. Myofilaments from CapZ mice lacked changes in CapZIP. Time to exhaustion was lower in CapZ mice in both swimming and running protocols. Actomyosin MgATPase activity was maintained in wildtype mice and impaired with CapZ deficiency. Exercise increased myofilament protein phosphorylation in wildtype mice but not in transgenic animals. Exercise‐dependent increases in myofilament PKC‐α and ‐ε were mitigated in CapZ mice. Telethonin/Tcap levels decreased significantly in CapZ‐deficient myofilaments with exercise, and leiomodin 2 increased in wildtype myofilaments. These data show Cardiac CapZ is a critical player in the physiological response to exercise and that CapZ‐actin binding is rapidly altered with exercise. Decreased cardiac CapZ limits exercise capacity, impairs myofilament regulation, and leads to a less stable contractile apparatus.

## Graphical abstract

CapZ regulates actin addition at the barbed ends of sarcomeric actin. At rest, phosphorylated CapZIP slows addition, and dephosphorylated CapZIP promotes a wobble state to allow actin cycling. During exercise, CapZIP is dephosphorylated to permit increased actin addition at the barbed ends. Increased leiomodin binding to cardiac myofilaments stabilizes filament structure during remodeling. Decreased CapZ diminishes regulation and prevents the stabilizing addition of leiomodin. CapZ is critical to maintaining myofilament function under physiological stress and maximizing exercise capacity.

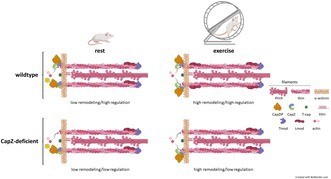

## 1 Introduction

The heart is a highly dynamic organ, responding rapidly to a range of environmental stimuli to match function with demand. For example, exercise increases heart rate and myocardial contractility within seconds of onset [1]. The period of time associated with the physical activity is described as the “acute phase,” and is characterized by a series of molecular changes that drive adaptations which have profound short‐ and long‐term implications on cardiac structure and function [2, 3]. Despite the acknowledged ability of the heart to respond quickly to exercise and the long‐term impact the acute molecular alterations have, there is limited understanding of the intracellular and molecular events that underlie the functional transformation of the myocardium during aerobic exercise [4, 5].

Chronic exercise regimens dramatically alter the architecture of the heart in large part through cellular hypertrophy. Much of the hypertrophic response involves alterations in gene expression that ultimately lead to both quantitative and qualitative proteomic changes. Although these structural alterations are chronic, taking weeks to reach equilibrium, there is evidence that the molecular structure of cardiac myocytes is affected much more rapidly. For example, physical stress applied to both skeletal [6] and cardiac [7] muscle rapidly alters actin filament capping by the Z‐disc protein CapZ, leading to actin filament remodeling. Although these studies were instrumental in identifying CapZ as a potential player in the acute response to exercise, intermittent stretching of muscle and the use of cultured cells fail to recapitulate the complex neural, humoral, immunological, and mechanical changes associated with aerobic exercise [3, 5]. Thus, despite the recognized importance of CapZ in cellular structure and function, its regulation under physiological conditions remains largely unexplored [8, 9].

Several intracellular regulators of cardiac CapZ have been identified, but their responses to physiological stressors like exercise have not been investigated. CapZ interacting protein (CapZIP) antagonizes the capping of filamentous actin by CapZ, an effect that is prevented when CapZIP is phosphorylated [6]. By contrast, the Hsc70‐BAG3 complex stabilizes actin binding by CapZ [10]. Eyers [6] showed that in skeletal muscle, the CapZIP regulatory system is rapidly activated by electrical stimulation and allows for skeletal muscle actin remodeling. Whether this regulatory system is similarly affected in cardiac muscle is not known, nor has the impact of exercise on Hsc70‐BAG3 been investigated.

It is unknown how cardiac myofilament remodeling is regulated during exercise, as previous studies have all examined changes post‐exercise. Given the central role of CapZ in contributing to the architecture of the thin filaments, knowledge of its regulation is crucial for understanding the mechanisms by which the heart responds to exercise. The objectives of this study were to determine how sarcomeric CapZ is impacted by acute exercise and to identify the underlying molecular mechanisms that regulate thin filament remodeling.

## 2 Materials and Methods

### 2.1 Statement of Ethics

All procedures were approved by and conducted in accordance with the guidelines set by the Animal Care and Use Committee of the University of Guelph (AUP #4406) and the Canadian Council on Animal Care.

### 2.2 CapZ Deficient Mice

Cardiac‐restricted CapZ‐deficient mice have been described previously [11, 12, 13, 14]. Z‐disc‐associated CapZ is decreased by ~20% through the overexpression of the β_2_‐subunit specifically in the heart [11]. A reduction of cardiac CapZ by > 20% is lethal [11]. CapZ‐deficient transgenic mice were homozygous for the transgene allele and were backcrossed to wildtype C57BL/6 mice for between 3 and 8 generations. All studies used female mice that were 8–12 weeks old. Wildtype mice were age, sex, and strain (C57BL/6) matched. Mice were group housed at the Central Animal Facility at the University of Guelph (room temperature 23°C) on a 12 h light/dark cycle and provided food and water ad libitum.

### 2.3 Exercise Fatigue Testing

Exercise tolerance was determined by swimming or running mice to exhaustion. For the swim test, mice were acclimated to the swimming bath with a 20 min swim exposure. Two days later, mice were weighed, and weights totalling 10% of body weight were attached to the tail. Mice were placed in a temperature‐controlled (30°C) water bath, and water was recirculated to stimulate swimming. Exhaustion was taken as the point when mice stopped swimming and sank to the bottom for ~5 s. For the running test, mice were acclimated with two 10 min exercise sessions (separate 2 days) on the treadmill at a 5% grade incline, paced at 15 m/min. Fatigue testing began with a treadmill speed of 12 m/min on an incline of 20%, and pacing was increased by 1 m/min at 2, 5, 10, and every subsequent 10 min [15]. Exhaustion was defined as an inability/refusal of the mouse to continue running when gently encouraged with a bottle brush.

### 2.4 Myofilament Isolation

Cardiac myofilaments were isolated as we have published [16, 17]. Briefly, hearts were homogenized in ice‐cold Standard Buffer (60 mM KCl, 30 mM Imidazole (pH 7.0), 2 mM MgCl_2_) containing phosphatase and protease inhibitors. The homogenate was centrifuged for 15 min at 12 000 g at 4°C. The resulting pellet was resuspended for 45 min in ice‐cold Standard Buffer plus 1% Triton X‐100 and centrifuged at 1100 g for 15 min at 4°C. Pellets were washed three times in ice‐cold Standard Buffer. Protein concentration was measured with a Bradford Protein Assay (Bio‐Rad Laboratories Ltd., Mississauga, ON, Canada).

### 2.5 Immunoblotting

Isolated cardiac myofilaments were subjected to SDS‐PAGE (12% separating gels) and transferred to nitrocellulose membranes for immunoblotting. Membranes were blocked in TBS containing 5% milk powder for 1 h at room temperature and then incubated with primary antibodies for CapZIP, phosphorylated CapZIP (S179), BAG3, PKC‐ε, actin (MilliporeSigma, Oakville, ON, Canada); Hsc70, leiomodin 2 (Santa Cruz Biotechnology, Dallas, TX, USA); PKC‐α, telethonin/Tcap (BD Biosciences, San Jose, CA, USA); tropomodulin 1 (Abcam Inc., Toronto, ON, Canada); and CapZ (Developmental Studies Hybridoma Bank, University of Iowa, Iowa City, IA, USA) overnight at 4°C. All primary antibodies were diluted 1:1000, except Hsc70, BAG3, TMOD1, Tcap (1:2000), and actin (1:25 000). Horseradish peroxidase‐conjugated secondary antibodies (anti‐mouse or anti‐rabbit, 1:5000, MilliporeSigma, Oakville, ON, Canada) were added for 1 h at room temperature. Protein bands were detected using Western Lightning (PerkinElmer Life and Analytical Sciences, Woodbridge, ON) and a Bio‐Rad ChemiDoc MP Imaging System (Bio‐Rad Laboratories Ltd., Mississauga, ON). Data were analyzed with ImageJ software (NIH, Bethesda, MD, USA). Immunoblot data was normalized to protein load as determined by Ponceau staining.

### 2.6 Myofilament Protein Phosphorylation

Myofilament proteins (10 μg) were separated using 12% SDS‐PAGE and fixed in 50% methanol‐10% acetic acid overnight at room temperature. Gels were stained with Pro‐Q Diamond phosphoprotein stain (Thermo Fisher Scientific, Mississauga, ON, Canada) according to the manufacturer's instructions to determine total protein phosphorylation. Gels were imaged using a Bio‐Rad ChemiDoc MP Imaging System (Bio‐Rad Laboratories Ltd., Mississauga, ON, Canada) and data were analyzed with ImageJ software (NIH, Bethesda, MD, USA). Total protein load was determined by Coomassie staining of the same gels, and total protein phosphorylation was normalized to total protein.

### 2.7 Myofilament Activity

Actomyosin MgATPase activity was determined using a modified Carter assay as we have published [16]. Isolated cardiac myofilaments (25 μg) were incubated in reaction buffers containing variable levels of free calcium. Activating (pCa 4.0) and relaxing (pCa 9.0) buffers were mixed to prepare the reaction buffers used. Myofilaments were incubated in reaction buffers for 10 min at 32°C, and the reactions were quenched with equal volumes of ice cold 10% trichloroacetic acid. The production of inorganic phosphate by ATP consumption was measured by adding 0.5% FeSO_4_ and 0.5% ammonium molybdate in 0.5 M H_2_SO_4_.

### 2.8 Statistical Analysis

The effect of exercise on CapZ regulation, myofilament function, protein phosphorylation, and exercise performance was analyzed using an unpaired Student's t‐test. All effects are compared to controls of the same animal group. Data are presented as mean ± SEM. *p* < 0.05 was selected to indicate significant differences.

## 3 Results

### 3.1 Acute Exercise Impacts Myofilament CapZ Regulation

**FIGURE 1:**
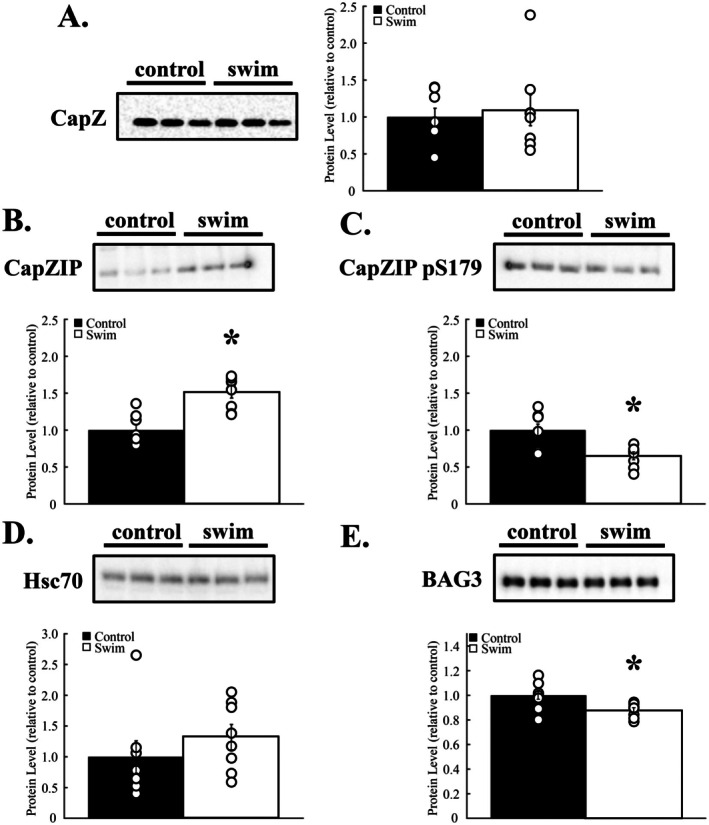
Cardiac CapZ regulation following acute exercise. Cardiac myofilaments were isolated after an acute bout of exercise and probed with immunoblotting. (A) Myofilament CapZ levels were not impacted by an acute bout of swimming. (B) CapZIP increased 51% ± 8% over control samples. (C) Phosphorylation of CapZIP at S179 decreased by 35% ± 5% compared to non‐exercise controls. (D) Myofilament‐associated Hsc70 levels were not altered by exercise. (E) BAG3 decreased by 12% ± 2% compared to controls. *N* = 9 in each group. Data are presented as mean ± SEM. *p < 0.05 versus control.

Cardiac myofilaments were isolated from mouse hearts following 20 min of swim exercise, and CapZ levels were measured by immunoblotting. Total myofilament CapZ protein levels were unaffected by exercise (Figure 1, *p* = 0.716).

CapZ can “wobble” when bound to actin by releasing the α‐subunit from actin and remaining attached to the thin filament through the C‐terminal tentacle of the β‐subunit [18, 19]. To determine if CapZ binding is affected by exercise, cardiac myofilaments from mice were probed with antibodies for the CapZ regulatory proteins CapZIP, Hsc70, and BAG3 following exercise. CapZIP protein increased 51% ± 8% over sedentary controls (Figure 1, *p* = 0.0003). Phosphorylation of myofilament‐associated CapZIP at S179 was significantly decreased by 35% ± 5% with exercise (*p* = 0.002). The increased levels of myofilament‐associated CapZIP coupled with increased phosphorylation result in a weakened interaction between CapZ and actin [6].

Hsc70 and BAG3 strengthen CapZ‐actin interactions [10]. Myofilament Hsc70 levels increased by 32% ± 20% with exercise; although this was not significantly different than controls (*p* = 0.323). The Hsc70 binding partner BAG3 decreased 12% ± 2% with exercise (*p* = 0.021). The lack of any coherent changes in Hsc70‐BAG3 levels indicates that this complex is unlikely to affect CapZ binding to sarcomeric actin.

### 3.2 Reduced Cardiac CapZ Shortens Time to Fatigue

**FIGURE 2:**
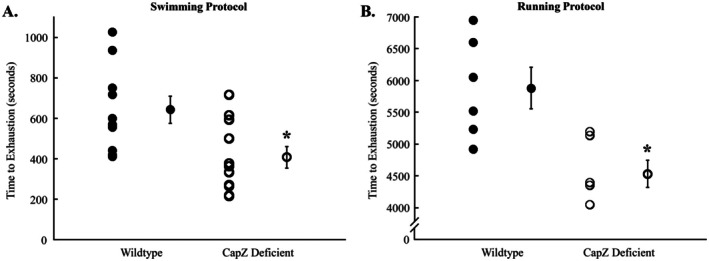
Time to exhaustion in wildtype and cardiac CapZ‐deficient mice. Mice were exercised to exhaustion using a (A) swimming or (B) running test. Average time to exhaustion for wildtype mice using the swimming protocol was 682 ± 78 s which was significantly longer than the 454 ± 65 s for CapZ‐deficient transgenic mice. The running protocol produced similar results with wildtype mice taking 5036 ± 396 s to reach exhaustion, compared to only 4531 ± 178 s for cardiac CapZ‐deficient transgenic mice. *N* = 10 in each group for swimming and *N* = 6 in each group for running. Data are presented as mean ± SEM. *p < 0.05 versus control of the same exercise.

To determine what role CapZ has in the acute response to exercise, we subjected mice that have an approximately 20% reduction in cardiac CapZ levels [12, 13] to exhaustive bouts of exercise. In a swimming protocol, the time to exhaustion for wildtype mice was 682 ± 78 s (Figure 2A). CapZ‐deficient transgenic mice had a significantly shorter time to exhaustion, with an average of 454 ± 65 s (*p* = 0.012). To determine if the effects on time to fatigue were specific to the swimming protocol, we subjected mice to a bout of exhaustive running. Similar to the swim test, in a running protocol, the time to exhaustion was 5879 ± 325 s for wildtype mice, which was significantly longer than the 4532 ± 210 s of cardiac CapZ‐deficient transgenic mice (Figure 2B, *p* = 0.006). The results demonstrate that a loss of myofilament‐associated CapZ impairs exercise performance and that the effects are not exercise‐specific.

### 3.3 Reduced CapZ Impacts CapZ Regulation

**FIGURE 3:**
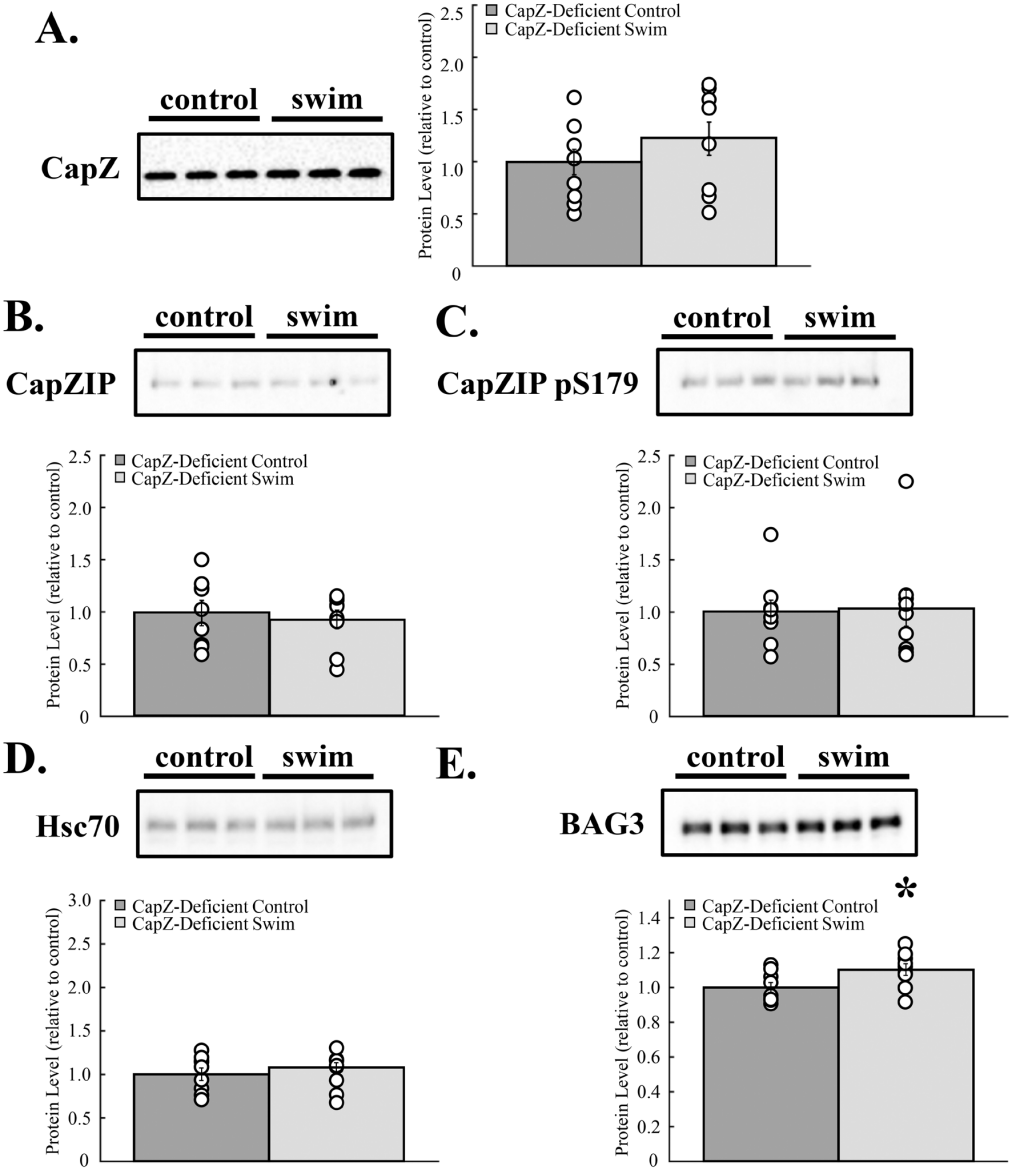
Cardiac CapZ‐deficient mice exhibit an impaired CapZ regulatory response to exercise. Cardiac myofilaments were isolated after an acute bout of exercise and probed with immunoblotting. Neither (A) CapZ, (B) CapZIP, (C) phosphorylation of CapZIP at S179, nor (D) myofilament‐associated Hsc70 levels were affected by exercise. (E) BAG3 increased by 10% ± 3% compared to controls. *N* = 9 in each group. Data are presented as mean ± SEM. *p < 0.05 versus control.

To determine if CapZ regulation during exercise was disrupted in cardiac CapZ‐deficient mice, cardiac myofilaments were probed for CapZ and key regulatory proteins using immunoblotting. CapZ (*p* = 0.285), CapZIP (*p* = 0.594), and Hsc70 (*p* = 0.400) levels were unchanged following exercise, as was the phosphorylation of CapZIP (*p* = 0.885) (Figure 3). Myofilament BAG3 levels increased 10% ± 3% following exercise (*p* = 0.031). Unlike wildtype mice, CapZ‐deficient transgenic mice lack a CapZ regulatory response to acute exercise, as measured by changes in myofilament CapZIP or Hsc70‐BAG3.

### 3.4 Reduced CapZ Alters Myofilament Functional Response to Exercise

**FIGURE 4:**
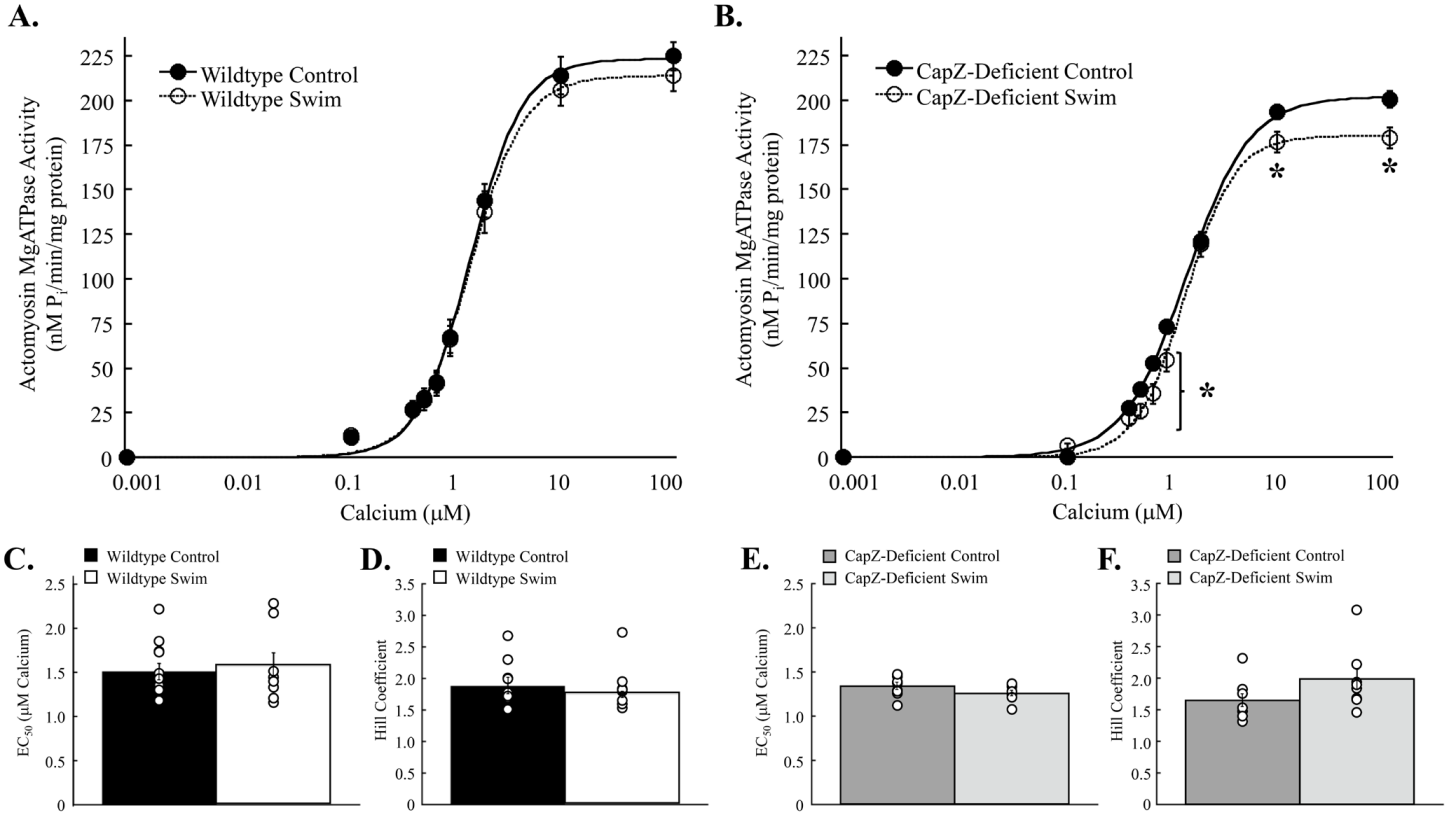
Myofilament actomyosin MgATPase activity with a single bout of exercise. Wildtype and cardiac CapZ‐deficient transgenic mice were subjected to a single bout of exercise and myofilament function assessed with an actomyosin MgATPase assay. (A) Actomyosin MgATPase‐calcium relationship was not altered with exercise in wildtype myofilaments. (B) Exercise decreased maximum actomyosin MgATPase activity in cardiac CapZ‐deficient transgenic mice from 223 ± 5 nM P_i_/min/mg protein to 199 ± 6 nM P_i_/min/mg protein. At free calcium concentrations below 1 μM actomyosin MgATPase activity was significantly impaired in cardiac CapZ‐deficient myofilaments. In wildtype myofilaments neither (C) EC_50_ nor (D) Hill coefficient were significantly impacted by exercise. Similarly, both (E) EC_50_ and (F) Hill coefficient were not significantly altered by exercise in cardiac CapZ‐deficient myofilaments. *N* = 9 in each group. Data are presented as mean ± SEM. *p < 0.05 versus control for same group. Coomassie, Coomassie stained gels for total protein load; MLC2, myosin light chain 2; MyBP‐C, myosin binding protein C; Phosphorylation, ProQ Diamond stained gels for total phosphorylation.

Cardiac myofilaments are the central contractile elements of the heart. Myofilament function was measured using actomyosin MgATPase activity following an acute bout of submaximal swimming. Myofilament function in wildtype hearts was not significantly affected by exercise (Figure 4A). Cardiac myofilaments from wildtype mice showed no change in maximum activation with exercise (*p* = 0.383), whereas CapZ‐deficient mice revealed a significant reduction in function at saturating levels of calcium (*p* = 0.012) and at several points of submaximal activation (< 1 μM free calcium; *p* = 0.001 to 0.029) (Figure 4B). EC_50_ values in wildtype (*p* = 0.767) and CapZ‐deficient (*p* = 0.164) were unaffected by exercise, as were Hill coefficients (wildtype, *p* = 0.424; CapZ‐deficient transgenic, *p* = 0.099). These data indicate that reduced CapZ impairs the ability of cardiac myofilaments to maintain function in the face of an exercise stressor.

**FIGURE 5:**
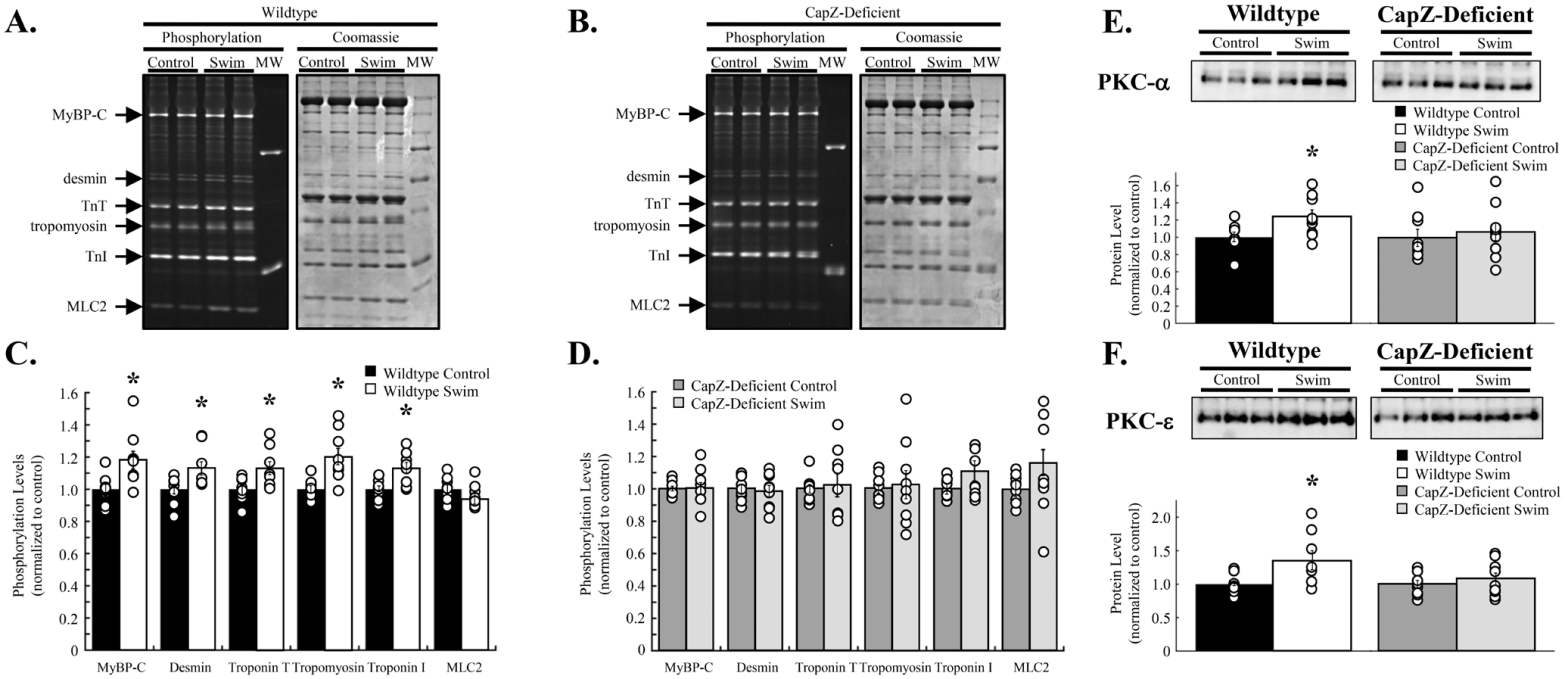
Changes in myofilament protein phosphorylation following a single bout of exercise. Following exercise cardiac myofilaments were resolved by SDS‐PAGE and stained for total phosphorylation. (A) Myofilaments from wildtype mice had significant increases in MyBP‐C, desmin, troponin T, tropomyosin, and troponin I, while MLC2 was unaltered. (B) By contrast there were no significant phosphorylation changes found in cardiac myofilaments from CapZ‐deficient transgenic mice. Summary results are shown in panels (C, D). Myofilament levels of PKC‐α increased after exercise in wildtype mice but not CapZ‐deficient transgenic mice. (E) Similarly, myofilament levels of PKC‐ε also increased in wildtype mice post‐exercise but not in CapZ‐deficient transgenic mice. Representative images of phosphorylation gels, Coomassie stained gels, and immunoblots are shown. *N* = 9 in each group. Data are presented as mean ± SEM. *p < 0.05 versus control for same group.

Phosphorylation of cardiac myofilaments permits a rapid response to sudden onset stressors like exercise. Cardiac myofilaments were resolved by SDS‐PAGE, and phosphorylation levels were probed with ProQ Diamond staining following a submaximal swimming protocol. Myofilaments from wildtype mice exhibited significant increases in total phosphorylation levels for myosin binding protein C (*p* = 0.011), desmin (*p* = 0.019), troponin T (*p* = 0.012), tropomyosin (*p* = 0.004), and troponin I (*p* = 0.003), whereas myosin light chain 2 (*p* = 0.154) was not significantly affected (Figure 5A,C). CapZ‐deficient myofilaments showed no significant changes in total phosphorylation (MyBP‐C, *p* = 0.976; desmin, *p* = 0.702; TnT, *p* = 0.744; tropomyosin, *p* = 0.785) although troponin I (*p* = 0.111) and myosin light chain 2 (*p* = 0.102) phosphorylation levels tended to rise (Figure 5B,D).

Cardiac myofilament phosphorylation is mediated by a number of different kinases. Work by us [12, 13, 14, 16, 17] and others [20, 21, 22, 23] has found that CapZ influences myofilament regulation by protein kinase C (PKC) and is a substrate for this family of kinases. To determine how exercise alters myofilament‐associated PKC and if a decrease in cardiac CapZ levels alters exercise‐dependent changes in sarcomeric PKC, we probed isolated cardiac myofilaments for PKC‐α (calcium‐dependent isoform) and PKC‐ε (calcium independent isoform). Whereas acute exercise increased both myofilament‐associated PKC‐α (*p* = 0.031) and ‐ε (*p* = 0.045) in wildtype hearts, the transgenic reduction in CapZ levels prevented these increases (Figure 5E,F; PKC‐α, *p* = 0.661; PKC‐ε, *p* = 0.454). These patterns of PKC activation may explain both the increase in myofilament protein phosphorylation following exercise in wildtype mice and the lack of change in CapZ‐deficient mice.

### 3.5 Reduced CapZ Destabilizes Sarcomeric Actin Filaments During Acute Exercise

**FIGURE 6:**
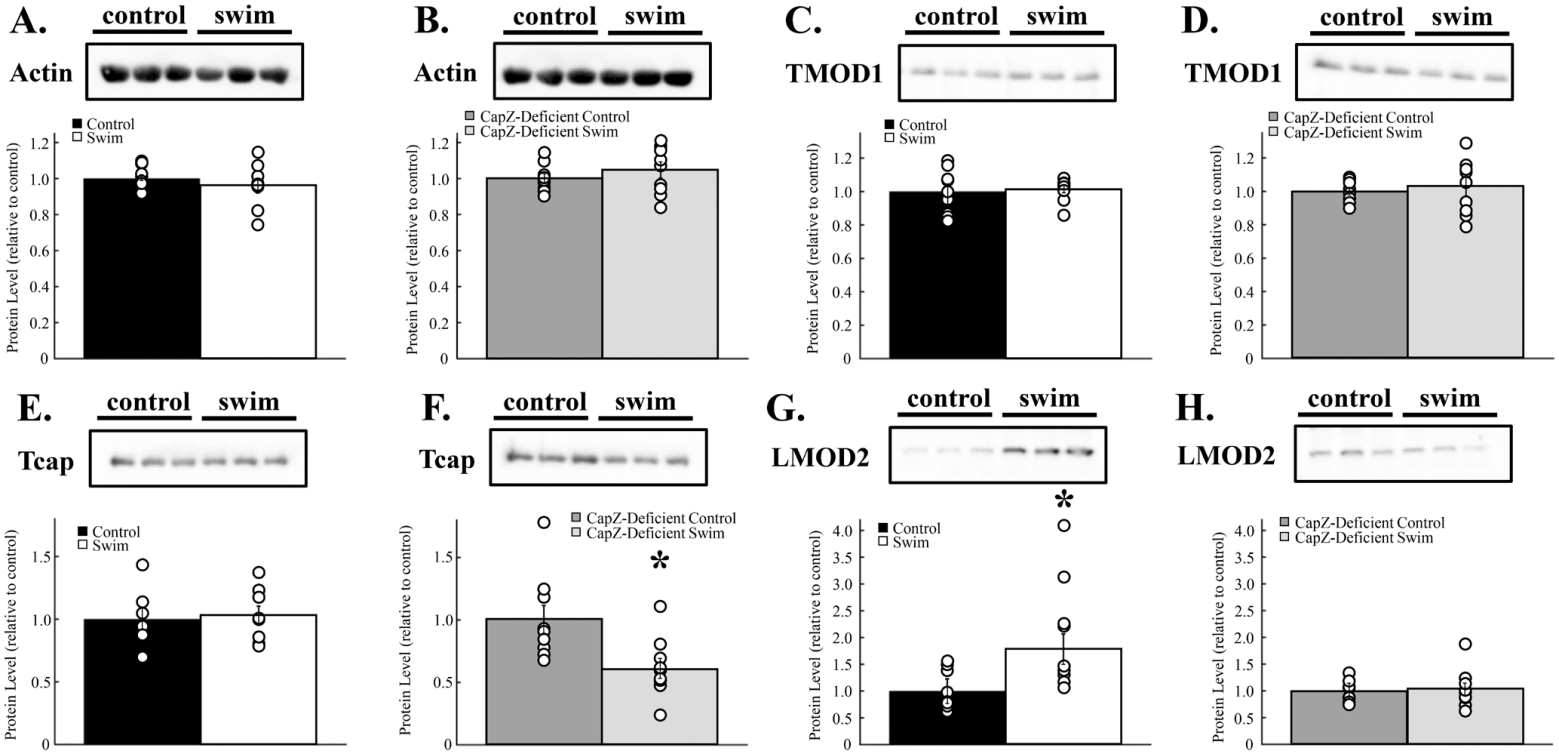
Myofilament‐binding proteins following acute exercise. Cardiac myofilaments were isolated after a single exercise session and subjected to immunoblotting. Sarcomeric actin levels were not altered by exercise in wildtype or cardiac CapZ‐deficient transgenic mice (A, B). Similarly, tropomodulin levels remained unaffected by exercise (C, D). Tcap/telethonin levels did not change with exercise in wildtype myofilaments (E) but did decrease by 39% ± 8% in CapZ‐deficient mice (F). Conversely, leiomodin levels rose 78% ± 28% in myofilaments from wildtype mice with exercise (G) but did not change in cardiac CapZ‐deficient mice (H). *N* = 9 in each group. Data are presented as mean ± SEM. *p < 0.05 versus control for same group. LMOD2, leiomodin 2; Tcap, telethonin; TMOD1, tropomodulin 1.

Exercise imposes a physical and neurohumoral stress on cardiac myocytes and demands a higher level of heart function. CapZ regulates both actin filament growth and acts as a physical anchor for the thin filaments at Z‐discs. To determine if the reduction in CapZ destabilizes the myofilaments under these stressors, we probed isolated cardiac myofilaments for anchoring and stabilizing proteins after an acute bout of swimming. Total sarcomeric actin was unaffected by submaximal exercise in both wildtype (*p* = 0.836) and CapZ‐deficient transgenic mice (*p* = 0.442) (Figure 6). The pointed‐end actin binding protein tropomodulin was also unaffected by exercise in both groups (wildtype, *p* = 0.933; CapZ‐deficient transgenic, *p* = 0.658). Levels of the titin capping protein telethonin/Tcap did not change in wildtype mice (*p* = 0.806) but significantly decreased by 39% ± 8% in CapZ‐deficient mice (*p* = 0.014). The actin‐binding protein leiomodin 2 increased 78% ± 28% in myofilaments from wildtype mice following acute exercise (*p* = 0.020) whereas myofilaments from CapZ‐deficient mice (*p* = 0.143) exhibited no significant changes in leiomodin 2 levels. Together, these results show that cardiac myofilament regulation by a variety of binding proteins is disrupted with a small decrease in CapZ levels.

## 4 Discussion

Exercise exerts a rapid stress on the heart that must be quickly matched to maintain performance and prevent cardiovascular collapse. The molecular basis of these changes is poorly characterized, in particular myofilament adaptations, limiting our understanding of how the heart responds to physiological stress. In this study, we identify the actin capping protein CapZ as a critical player in the cardiac response to exercise. CapZ binding to sarcomeric actin is weakened during exercise, and yet its reduction limits exercise capacity in mice, indicating a finely controlled remodeling of the thin filaments. The presence of CapZ during exercise is necessary to maintain normal myofilament function in the face of increased stress, as demonstrated by a decrease in exercise performance when CapZ expression is reduced. Finally, a decrease in CapZ expression disrupts the binding of several critical myofilament‐associated proteins during exercise, like Tcap/telethonin and leiomodin, which help to maintain sarcomeric integrity. Together, this study represents the first investigation of molecular changes in cardiac myofilaments during an acute bout of exercise and discovers a critical role for CapZ in the myofilament adaptation to exercise.

CapZ exists in a “wobble” state with actin in which the α‐subunit of the capping protein dynamically binds and dissociates from actin, remaining bound only through the β‐subunit [18]. In situations like cell motility where actin uncapping periods may be prolonged to allow for sustained filamentous growth, the complete dissociation of CapZ can occur. But in striated muscle where prolonged periods of unrestrained actin addition could interfere with contractile function, this model is not likely. An analogous situation has been described for the pointed‐end capping protein tropomodulin in which actin depolymerization is permitted without the dissociation of tropomodulin from the thin filament [24]. Our data show that during exercise, CapZ does not leave the myofilament compartment. However, the presence of increased levels of CapZIP would favor a disruption of the CapZ‐actin binding. Based on these data, we propose a model of CapZ regulation during exercise in which actin capping by CapZ is weakened by CapZIP. This weakening comes in the form of favoring the open wobble state where CapZ remains bound to actin through the β‐subunit, but the uncapping is sufficient to allow for actin additions. We found no change in the levels of myofilament actin during exercise, which suggests that actin filament lengths are maintained during exercise and that the weakening of CapZ allows for increased treadmilling of actin. Lin and colleagues [22] showed that mechanical perturbation of neonatal cardiac myocytes meant to mimic exercise induces a rapid increase in actin dynamics, which provides support for our model.

The impact of exercise training on the heart is well known. By contrast, the myofilament alterations that drive the myocardial response to acute exercise have not been widely investigated. Muller et al. [25] found that a single bout of acute exercise increased passive stiffness of isolated cardiac myocytes through a complex alteration in titin phosphorylation. Although an increase in myofilament stiffness can contribute to diastolic dysfunction, the acute changes in stiffness that occur in exercise were hypothesized to improve cardiac output by supporting the Frank‐Starling mechanism of increased contractility [25]. Chakouri and colleagues [26] examined post‐translational changes in cardiac myofilaments subjected to exercise and found that a submaximal stress increased troponin I and myosin binding protein C phosphorylation at protein kinase A‐preferred sites. Interestingly, these myofilament alterations did not impact myofilament activation as assessed by force‐calcium curves. In our study, we found a similar increase in myofilament protein phosphorylation and extended these changes to include desmin, troponin T, and tropomyosin, but not myosin light chain 2. Eikemo et al. [27] showed that the rate of myosin light chain 2 phosphorylation is slow, increasing by ~1.2% per minute when the heart is stimulated, but only when phosphatase activity was inhibited. The relatively slow rate of phosphorylation suggests that myosin light chain 2 phosphorylation is unlikely to significantly change during the acute events reported here. We also found no significant changes in myofilament activation as quantified by an actomyosin MgATPase assay. Although there were no changes in myofilament function detectable in association with acute exercise, this does not preclude an important role for cardiac myofilaments. Like the titin phosphorylation changes reported by Muller and colleagues [25], the increase in desmin phosphorylation we observed may provide physical support for the stress of exercise. Furthermore, the oxidative stress of exercise has negative effects on cardiac myofilaments, and these changes may be countered by phosphorylation [26]. In this scenario, the increase in phosphorylation may offset the detrimental effects of myofilament oxidation, helping to maintain contractility.

Acute exercise applies a sudden stress to the heart, necessitating a rapid response. Myofilament protein phosphorylation allows for a quick and powerful response to stressors. These changes are mediated by protein kinases and phosphatases that have a number of potential targets within the contractile complex. Muller [25] reported PKC‐dependent increases in titin phosphorylation, which they ascribed to increased PKC‐α activity, although they did not determine if PKC‐α binding to cardiac myofilaments was altered with exercise. We have previously shown that CapZ is critical for PKC‐dependent control of cardiac myofilaments, and studies from Russell's group showed that CapZ binding is itself regulated by PKC‐ε [23, 28]. In this study, we show for the first time that myofilament‐associated PKC‐α and ‐ε increase during submaximal exercise and that these changes are mitigated in mice with a deficiency of cardiac CapZ. The inability of CapZ‐deficient transgenic mice to maintain physical activity for as long as wildtype may, in part, be explained by the disruption in PKC signaling. Without PKC‐α myofilament stiffness driven by titin phosphorylation would be reduced, and the Frank‐Starling effect would be impaired, while a lack of increased PKC‐ε would impair CapZ regulation and thin filament remodeling.

Our data show that an acute bout of exercise is associated with a significant increase in cardiac myofilament‐associated leiomodin. Leiomodin is a known actin nucleator that co‐localizes with tropomodulin at the pointed ends of actin filaments. Our data show no significant increase in sarcomeric actin, which makes actin filament elongation unlikely, but a model of actin treadmilling proposed by Skwarek‐Maruszewska and colleagues [29] may explain enhanced myofilament leiomodin in the absence of increased sarcomeric actin. In this model, increased contractility stimulates leiomodin to bind to actin filaments near the Z‐disc and remain bound as actin treadmills through the filament. Leiomodin binding stabilizes the thin filament structure and maintains contractility in the face of an increase in demand [29, 30]. Interestingly, Szatmári and colleagues [30] report a reduction in actin‐activated MgATPase activity with the addition of leiomodin, which we did not observe in our experiments. Among the important differences between these works was the use of purified actin, myosin, and tropomyosin from a mixture of skeletal and cardiac muscle in the assays conducted by Szatmári et al. While it is possible that differences between the skeletal and cardiac muscle protein isoforms could yield different outcomes in terms of MgATPase activity and the impact of leiomodin, our use of intact and troponin‐regulated myofilaments may offer countering effects of the leiomodin inhibition to explain these discrepancies. The simultaneous changes in myofilament protein phosphorylation we observed—several of which are known to alter myofilament activation—could act as offsetting alterations to the inhibitory effects of leiomodin. Despite the noted inhibitory effects of leiomodin on MgATPase activity, we speculate that the ability of leiomodin to stabilize the dynamically changing myofilaments in exercising wild‐type mice, but not CapZ‐deficient transgenic mice, could contribute to the enhanced performance of wild‐type mice over their CapZ‐deficient counterparts.

In contrast to the significant changes in leiomodin that occur with exercise, myofilament Tcap/telethonin levels were unaffected in wildtype mice but reduced in CapZ‐deficient transgenic mice. Chronic reductions in Tcap/telethonin are associated with both hypertrophic and dilated cardiomyopathy, possibly as a result of problems with mechanosensing and alterations in intracellular signaling cascades [31]. However, the functional impact of acute Tcap/telethonin alterations, as we report here, is not known. Tcap/telethonin knockout mice have no detectable impairments in cardiac function at rest, but the application of stress triggers intracellular pathways that drive heart failure [32]. We can speculate that the maintained levels seen in wildtype mice, contrasted with the decrease in CapZ‐deficient transgenic mice subjected to exercise, and the correlation with exercise performance suggest an important role for Tcap/telethonin in the response to exercise similar to that seen under pathological conditions. But the multitude of changes that occur simultaneously during exercise precludes a definitive conclusion. The acute regulation of Tcap/telethonin should be investigated further to provide a physiological complement to what is already known about its pathological role.

**FIGURE 7:**
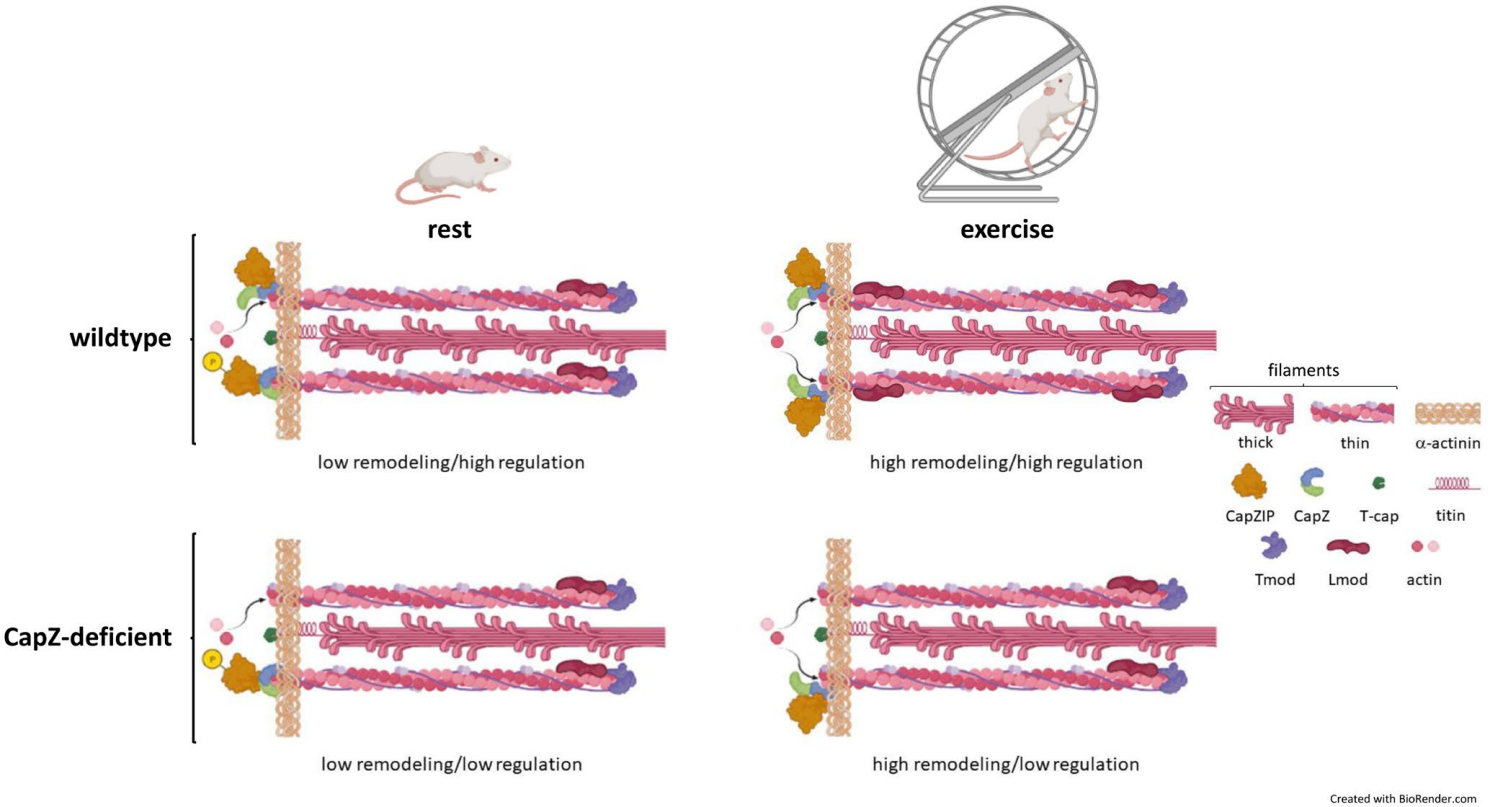
Cardiac CapZ regulation during acute exercise. CapZ regulates actin addition at the barbed ends of sarcomeric actin. At rest phosphorylated CapZIP slows addition and dephosphorylated CapZIP promotes a wobble state to allow actin cycling. During exercise CapZIP is dephosphorylated to permit increased actin addition at the barbed ends. Increased leiomodin binding to cardiac myofilaments stabilizes filament structure during remodeling. Decreased CapZ diminishes regulation and prevents the stabilizing addition of leiomodin. CapZ is critical to maintain myofilament function under physiological stress and maximizing exercise capacity.

Cardiac sarcomeres are often viewed as static structures in terms of their physical composition. Their constant activation and relaxation with each beat of the heart demand that the structures are constantly functioning. While it is likely that individual sarcomeres are repaired, remodeled, or even removed while the heart is functioning, the environment demands that these processes be undertaken as neighboring sarcomeres continue to function. We propose a role for CapZ in myofilament maintenance during exercise in which the Z‐disc protein is moved from the closed to open state to allow for thin filament remodeling (Figure 7). Our data show that during acute exercise several CapZ‐regulatory elements are altered, modifying CapZ in a way that would promote the wobble state and permit actin modifications. This dynamic fluctuation creates a window of opportunity to remodel or repair sarcomeres, without significantly disrupting the physical architecture of the contractile apparatus. Thin and thick filament‐associated proteins like leiomodin stabilize the filaments as actin monomers are added and removed. Such a highly coordinated process is necessary to mediate alterations without impairing function. In our transgenic mouse models where the levels of CapZ are reduced, the myofilament structure is not grossly disrupted [33]. However, more subtle molecular changes like disruptions in leiomodin binding to cardiac myofilaments disrupt the coordinated regulation of cardiac myofilaments during exercise by failing to fully stabilize actin filaments.

In short, our data show that CapZ is a dynamically regulated cardiac Z‐disc protein whose function is critical to ensuring optimal exercise performance, and that small disruptions in the expression of the protein can significantly impact cardiac function such as to limit exercise capacity. We further extend our earlier work in which we found a role for CapZ in regulating cardiac myofilament function [12, 13, 14, 16, 17, 34] and show here that the role of CapZ extends beyond its structural role [11] and into a role as a key player in the response to physiological stress. Understanding the role of CapZ in shaping the myocardial response to physiological and pathological stress is critical to differentiating between the adaptive and maladaptive mechanisms that characterize exercise training and disease, respectively. Insight into the mechanistic underpinnings of exercise offers a unique opportunity to target these pathways therapeutically to produce beneficial effects in the heart and to potentially mitigate disease.

## Author Contributions

Logan K. Townsend conducted running experiments. David Wright contributed to the design of the running study. W. Glen Pyle conducted the swimming study, assisted with the running experiments, as well as collecting the myofilament ATPase data, protein phosphorylation data, and immunoblotting. All authors contributed to the writing and editing of the manuscript.

## Conflicts of Interest

The authors declare no conflicts of interest.

## Supporting information


**Figure S1:** fsb270950‐sup‐0001‐FigureS1.pdf.

## Data Availability

The data that support the findings of this study are available in the Materials and Methods, and Results sections of this article.
