# Supplementary figures and images for: Cardiac CapZ Regulation During Acute Exercise in Female Mice
Source: FASEB J. 2025 Aug 20;39(16):e70950. doi: 10.1096/fj.202502431R (PMC12365864; doi:10.1096/fj.202502431R)

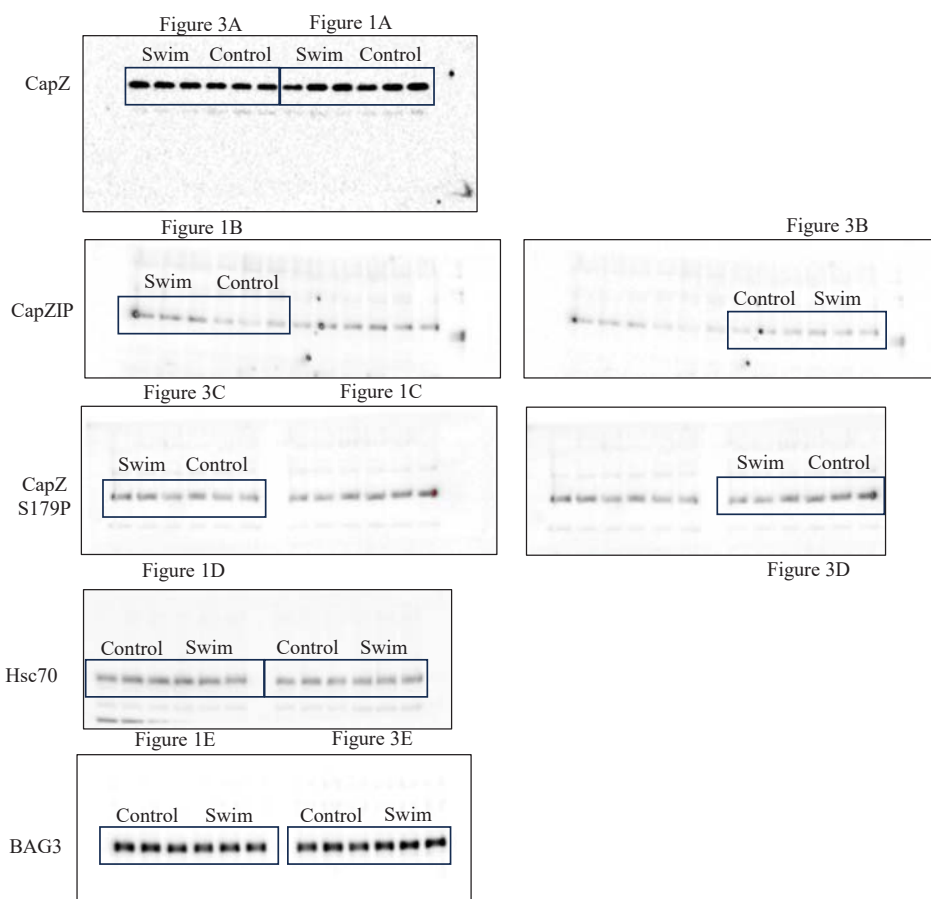

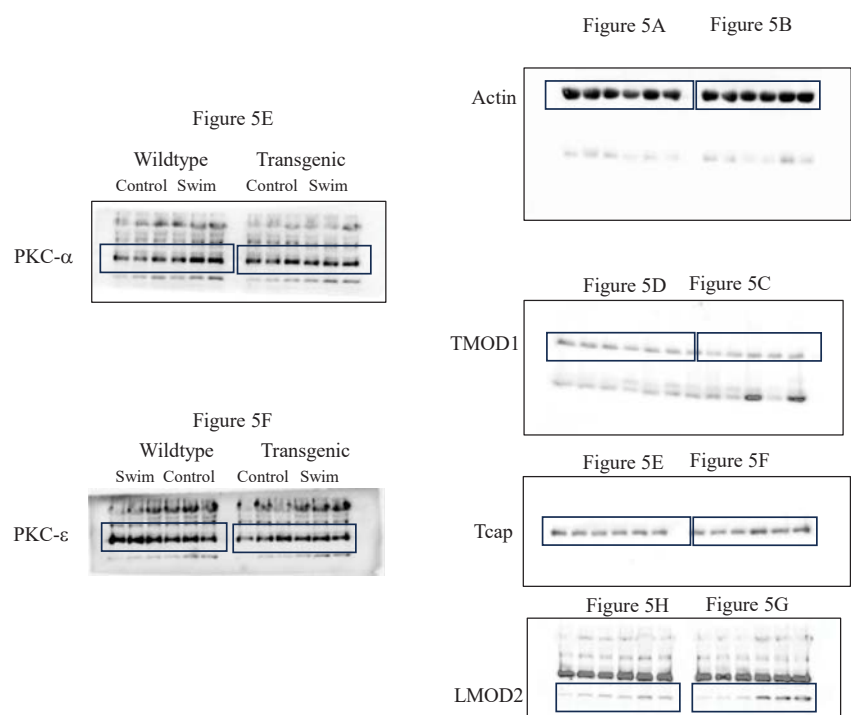

Supplement: Supplementary file 1 — Figure S1: fsb270950‐sup‐0001‐FigureS1.pdf. [file FSB2-39-e70950-s001.pdf]
